# Ethnic inequities in the patterns of personalized care adjustments for ‘informed dissent’ and ‘patient unsuitable’: a retrospective study using Clinical Practice Research Datalink

**DOI:** 10.1093/pubmed/fdad104

**Published:** 2023-07-11

**Authors:** Brenda Hayanga, Mai Stafford, Mark Ashworth, Jay Hughes, Laia Bécares

**Affiliations:** Department of Global Health and Social Medicine, King’s College London, London WC2B 4BG, UK; The Health Foundation, London EC4Y 8AP, UK; Department of Population Health Sciences, King’s College London, London SE1 1UL, UK; The Health Foundation, London EC4Y 8AP, UK; Department of Global Health and Social Medicine, King’s College London, London WC2B 4BG, UK

**Keywords:** ethnicity, primary care, personalized care adjustments, quality

## Abstract

**Background:**

In England, general practitioners voluntarily take part in the Quality and Outcomes Framework, which is a program that seeks to improve care by rewarding good practice. They can make personalized care adjustments (PCAs), e.g. if patients choose not to have the treatment/intervention offered (‘informed dissent’) or because they are considered to be clinically ‘unsuitable’.

**Methods:**

Using data from the Clinical Practice Research Datalink (Aurum), this study examined patterns of PCA reporting for ‘informed dissent’ and ‘patient unsuitable’, how they vary across ethnic groups and whether ethnic inequities were explained by sociodemographic factors or co-morbidities.

**Results:**

The odds of having a PCA record for ‘informed dissent’ were lower for 7 of the 10 minoritized ethnic groups studied. Indian patients were less likely than white patients to have a PCA record for ‘patient unsuitable’. The higher likelihood of reporting for ‘patient unsuitable’ among people from Black Caribbean, Black Other, Pakistani and other ethnic groups was explained by co-morbidities and/or area-level deprivation.

**Conclusions:**

The findings counter narratives that suggest that people from minoritized ethnic groups often refuse medical intervention/treatment. The findings also illustrate ethnic inequities in PCA reporting for ‘patient unsuitable’, which are linked to clinical and social complexity and should be tackled to improve health outcomes for all.

## Background

The Quality and Outcomes Framework (QOF) is a pay-for-performance scheme in England, which rewards practices for the delivery of evidence-based standards of care.^1^^,^^2^ To safeguard patients from inappropriate care and/or prevent practices from being penalized for not achieving targets for reasons beyond their control, the scheme allows general practitioners (GPs) to make personalized care adjustments (PCAs) to exclude patients from performance indicators.^2^^,^^3^ Patients can be exempt from performance targets if a service is unavailable or if a patient is newly registered, newly diagnosed, unsuitable for treatment, does not respond to offers of care or refuses treatment (‘informed dissent’).^3^ Exemption from quality indicators is associated with health outcomes, including higher mortality risk and poor control of risk factors.^4–6^

PCA rates vary between practices, across quality indicators, health conditions and reasons for exemption. Exemptions are more prevalent in disadvantaged groups.^4^ Higher rates of ‘informed dissent’ are found in practices with a high number of registered patients, in socioeconomically deprived catchments, and fail to secure maximum renumeration in the previous year.^7^ Patients who are older, have multiple long-term conditions (MLTCs) and live in deprived areas are more likely to be removed from achievement calculations for being unsuitable or because of ‘informed dissent’.^6^ People from minoritized ethnic groups^8^ are overrepresented in domains that are more likely to be exempted from QOF indicators. Minoritized ethnic groups (with the exception of people of Indian, Chinese, white Irish and white other ethnicity) are more likely to be living in the most overall deprived 10% of neighborhoods in England in 2019.^9^ Older people from minoritized ethnic groups (with the exception of Black African men and Chinese people) have as many or more long-term conditions as white British people.^10^ However, the evidence from the few studies that have explored ethnic variations is unclear. One study, focused on patients with diabetes, reports that Black and South Asian patients have higher odds of being excluded from the HbA1c indicator when compared to white patients.^4^ An ecological analysis found lower rates of PCA among minoritized ethnic groups with asthma when compared to their white counterparts.^1^

The aim of this study is to assess whether there are ethnic inequities in PCA reporting. We focus on two PCA reasons: ‘patient unsuitable’ (exempted by a GP for a range of reasons, including failure to respond to maximum dose of treatment, adverse reaction to treatment, extreme frailty or supervening condition^11^^,^^12^) and ‘informed dissent’ (where patients do not agree to treatment or medical investigation). This will give us insight into the clinical judgments made by GPs and patient’s choice. Identifying groups that are not included in the QOF scheme can inform strategies to ensure that those who are eligible receive the recommended standard of care.

## Methods

### Data source and population

We conducted a retrospective cohort study using data from the Clinical Practice Research Datalink (CPRD) Aurum that contains longitudinal, routinely collected electronic health records from patients in general practice.^13^ The CPRD Aurum is representative of the population in England in terms of geographical spread, area-level deprivation, age and gender. In March 2022, the CPRD Aurum had ~13 million patients who were registered at currently contributing practices.^14^ We analyzed the CPRD Aurum data linked to ONS data to allow for the measurement of area-level deprivation using the Index of Multiple Deprivation (IMD)^15^ (based on patient’s residential Lower Layer Super Output Area) and Hospital Episode Statistics (HES) to improve the completeness of ethnicity recording. We drew upon a random sample of 690 000 patients aged ≥18 years on 1 January 2016.

### Measures

We extracted the ethnic identity from the Systemized Nomenclature of Medicine codes recorded by the GP.^16^^,^^17^ When ethnicity data were missing/incomplete, we drew this from the HES records. We used the England 2011 census to define ethnic categories, but we combined white British, white Irish and Other white because these separate categories were unavailable in the HES. We included all QOF long-term conditions for which there were the options of the two PCA codes of interest: ‘informed dissent’ and ‘patient unsuitability’ (Supplementary Table 1). We identified relevant PCA codes between 1 January 2016 and 31 December 2019 for all patients with the included QOF conditions. Other sociodemographic data comprised age, gender and deprivation. Socioeconomic deprivation was derived from the IMD quintiles based on the lower super output area boundaries of the patient’s address (Quintile 1 represents the least deprived).^15^

### Statistical analysis

We created an analytical sample that included only those with at least 1 of the 12 QOF conditions at baseline (1 January 2016) and complete data on age, gender, ethnicity and area-level deprivation. Men, people aged <45 years and those with one QOF condition were overrepresented in those with missing ethnicity data (Supplementary Table 2). We built separate logistic regression models for each outcome^18^ and included (i) each covariate separately; (ii) ethnicity, age and gender; (iii) ethnicity, age, gender and multiple QOF conditions and (iv) ethnicity, age, gender, multiple QOF conditions and IMD score. We also conducted a sensitivity analysis by creating separate models for men and women. We used RStudio (R04.2.0) for all our analyses.^19^

## Results

**Fig. 1:**
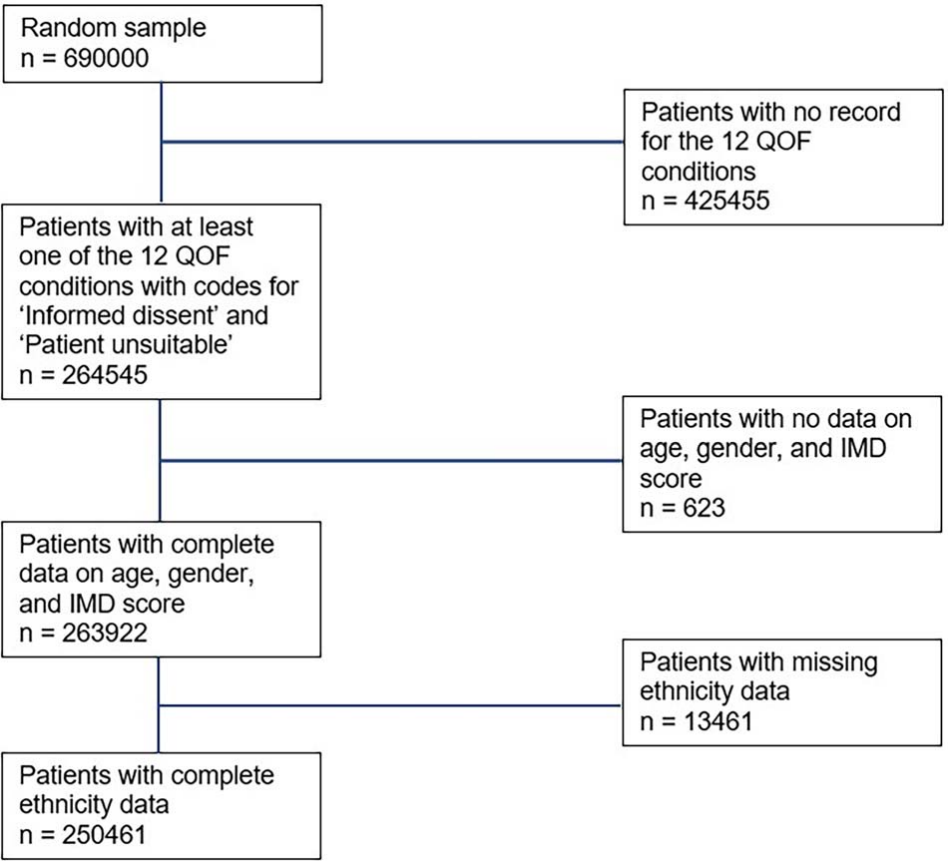
Flow chart to get analytical sample.

The number of patients with at least one QOF condition and complete data on ethnicity, gender, IMD score and age was 250 461 (Fig. 1). The majority were of white ethnicity (89.2%) (Table 1 and Supplementary Table 3). The Black Caribbean ethnic group had the highest proportion of people aged over 75 years (22%) and multiple QOF conditions (40.9%). Approximately, 50% of patients from Bangladeshi, mixed and Black Other ethnic groups were <45 years of age (Supplementary Table 3). Over 40% of patients from Bangladeshi, Black African, Black Caribbean, Black Other and Pakistani ethnic groups were living in the most deprived IMD quintile (Table 1).

**Table 1:** Sociodemographic characteristics and percentage of PCA by ethnic group

	*n*	% Women	% >75 years	% With multiple QOF conditions	% Living in IMD 5	% With informed dissent recorded	% With patient unsuitable recorded
Bangladeshi	1253	49.8	7.6	32.5	50.4	6.5	3.4
Black African	3652	53.3	6.2	26.0	45.8	6.2	3.0
Black Caribbean	3737	56.5	22.0	40.9	42.7	6.1	4.4
Black Other	1162	54.0	6.1	26.1	41.5	7.3	3.5
Chinese	741	56.4	12.6	24.2	16.5	6.5	2.0
Indian	5309	50.9	13.3	38.2	19.2	6.5	2.9
Mixed	2900	56.3	7.0	25.0	30.7	7.0	2.8
Other Asian	3035	51.0	8.7	31.2	18.8	6.3	3.1
Other	2154	49.8	10.0	25.2	26.5	5.4	3.5
Pakistani	3221	49.9	8.2	34.7	40.9	8.9	3.4
White	223 297	54.6	20.6	36.0	17.5	8.0	3.7

Across all ethnic groups, patients were more likely to receive a PCA record for ‘informed dissent’ than for ‘patient unsuitable’. While Pakistani patients had the highest proportions of people with a PCA record for ‘informed dissent’ (8.9%), patients of other ethnicity had the lowest (5.4%). By contrast, Black Caribbean people had the highest proportion of people with a PCA record for ‘patient unsuitable’ (4.4%), and Chinese patients had the lowest (2%) (Table 1).

### ‘Informed dissent’ PCA findings

In simple logistic regression analysis (Table 2), women were less likely to have a PCA record for ‘informed dissent’ than men [odds ratio (OR): 0.81; 95% confidence interval (CI): 0.79–0.83]. Patients aged 45–59 years and 60–74 years were more likely to have a PCA record for ‘informed dissent’ than patients younger than 45 years (OR: 1.34, 95% CI: 1.29–1.39 and OR: 1.1, 95% CI: 1.05–1.14, respectively). People with multiple QOF conditions were 2.21 times more likely to have a PCA record for ‘informed dissent’ than people with one QOF condition (95% CI: 2.14–2.27). Patients living in the most deprived quintile were 1.27 times more likely to have a PCA record for ‘informed dissent’ (95% CI: 1.22–1.33) than patients living in the least deprived quintile. In multiple logistic regression analysis, the association between ‘informed dissent’ and the above covariates followed a similar trend with the exception of age categories, where the association was in the opposite direction for people aged 60–74 years and ≥75 years. They were less likely to have a PCA record for informed dissent when compared to those aged ≤44 years (OR: 0.78, 95% CI: 0.75–0.81 and OR: 0.63, 95% CI: 0.60–0.66) (Table 3, Model 3).

**Table 2:** Simple logistic regression models showing the association between having a PCA record for ‘informed dissent’ and demographic characteristics

	ORs	95% CI	*P*
Ethnicity			
White	1		
Bangladeshi	0.8	0.63–0.99	**0.05**
Black African	0.76	0.67–0.87	**<0.001**
Black Caribbean	0.75	0.66–0.86	**<0.001**
Black Other	0.91	0.72–1.13	0.40
Chinese	0.8	0.59–1.06	0.13
Indian	0.8	0.71–0.89	**<0.001**
Mixed	0.87	0.75–1.00	0.06
Other Asian	0.77	0.67–0.89	**0.001**
Other	0.66	0.55–0.79	**<0.001**
Pakistani	1.13	1.00–1.28	**0.05**
Gender			
Men	1		
Women	0.81	0.79–0.83	**<0.001**
Age			
^*^<45	1		
45–59	1.34	1.29–1.39	**<0.001**
60–74	1.1	1.05–1.14	**<0.001**
75+	1	0.95–1.04	0.85
Multiple QOF conditions			
1 Condition	1		
2+ Conditions	2.21	2.14–2.27	**<0.001**
Area-level deprivation			
Least deprived Quintile 1	1		
Quintile 2	1.01	0.97–1.06	0.59
Quintile 3	1.07	1.02–1.12	**0.01**
Quintile 4	1.13	1.08–1.18	**<0.001**
Quintile 5	1.27	1.22–1.33	**<0.001**

Observations = 250 461.

The values in bold are *p* values that are less than 0.05.

**Table 3:** Multiple logistic regression models showing the association between having a PCA record for ‘informed dissent’ and demographic characteristics

	Model 1			Model 2			Model 3		
	ORs	95% CI	*P*	ORs	95% CI	*P*	ORs	95% CI	*P*
Ethnicity									
White	1			1			1		
Bangladeshi	0.79	0.62–0.98	**0.04**	0.74	0.58–0.92	**0.01**	0.69	0.55–0.87	**0.002**
Black African	0.73	0.63–0.83	**<0.001**	0.75	0.65–0.85	**<0.001**	0.7	0.61–0.81	**<0.001**
Black Caribbean	0.74	0.64–0.84	**<0.001**	0.71	0.62–0.81	**<0.001**	0.67	0.58–0.76	**<0.001**
Black Other	0.89	0.71–1.10	0.3	0.89	0.71–1.10	0.3	0.85	0.67–1.05	0.15
Chinese	0.8	0.59–1.06	0.13	0.88	0.65–1.17	0.4	0.88	0.65–1.17	0.39
Indian	0.78	0.70–0.87	**<0.001**	0.75	0.67–0.84	**<0.001**	0.74	0.66–0.83	**<0.001**
Mixed	0.88	0.76–1.01	0.08	0.88	0.76–1.02	0.09	0.86	0.74–0.99	**0.04**
Other Asian	0.75	0.65–0.87	**<0.001**	0.75	0.65–0.87	**<0.001**	0.74	0.64–0.86	**<0.001**
Other	0.65	0.53–0.77	**<0.001**	0.68	0.56–0.81	**<0.001**	0.66	0.55–0.80	**<0.001**
Pakistani	1.11	0.98–1.25	0.1	1.05	0.93–1.19	0.41	1.01	0.89–1.14	0.93
Gender									
Men	1			1			1		
Women	0.81	0.79–0.84	**<0.001**	0.81	0.79–0.83	**<0.001**	0.81	0.79–0.83	**<0.001**
Age									
^*^<45	1			1			1		
45–59	1.34	1.286–1.39	**<0.001**	1.13	1.08–1.17	**<0.001**	1.13	1.09–1.18	**<0.001**
60–74	1.08	1.038–1.13	**<0.001**	0.77	0.74–0.80	**<0.001**	0.78	0.75–0.81	**<0.001**
75+	0.99	0.948–1.04	0.68	0.62	0.59–0.64	**<0.001**	0.63	0.60–0.66	**<0.001**
Multiple QOF conditions									
1 Condition				1			1		
2+ Conditions				2.55	2.47–2.63	**<0.001**	2.52	2.44–2.60	**<0.001**
Area-level deprivation									
Least deprived Quintile 1							1		
Quintile 2							1	0.96–1.05	0.86
Quintile 3							1.05	1.00–1.10	0.05
Quintile 4							1.1	1.05–1.15	**<0.001**
Quintile 5							1.21	1.15–1.26	**<0.001**

Observations = 250 461. Model 1 adjusted for age and gender. Model 2 adjusted for age, gender and multiple QOF conditions. Model 3 adjusted for age, gender, multiple QOF conditions and area-level deprivation.

The values in bold are *p* values that are less than 0.05.

In the unadjusted and fully adjusted models, there were lower odds of having a PCA record for ‘informed dissent’ for Bangladeshi, Black African, Black Caribbean, Indian, Other Asian and other ethnic group people (Tables 2 and 3). People of Pakistani ethnicity were more likely to have a PCA record for ‘informed dissent’ than people of white ethnicity (OR: 1.13, 95% CI: 1.00–1.28) (Table 2). This association was no longer evident after adjusting for the sociodemographic characteristics in the other models (Table 3). Ethnic differences for ‘informed dissent’ were similar for men and women (Supplementary Tables 4 and 5).

### ‘Patient unsuitable’ PCA findings

In a simple logistic regression analysis, older people were more likely to have a PCA record for ‘patient unsuitable’ (Table 4). Those with multiple QOF conditions were 4.69 times more likely to have a PCA record for ‘patient unsuitable’ than those with one condition (95% CI: 4.48–4.91). Also, people living in the fifth most deprived quintile were 1.25 times more likely to have a PCA record for ‘patient unsuitable’ than those in the least deprived quintile (95% CI: 1.04–1.19). A similar pattern was observed in the multiple logistic regression analysis, albeit with some attenuation to the effect sizes (Table 5). In these models, women were less likely to have a PCA record for ‘patient unsuitable’ than men.

**Table 4:** Simple logistic regression models showing the association between having a PCA record for ‘patient unsuitable’ and demographic characteristics

	ORs	95% CI	*P*
Ethnicity			
White	1		
Bangladeshi	0.92	0.67–1.24	0.61
Black African	0.79	0.65–0.96	**0.02**
Black Caribbean	1.19	1.01–1.39	**0.03**
Black Other	0.95	0.68–1.28	0.75
Chinese	0.54	0.31–0.86	**0.02**
Indian	0.77	0.65–0.90	**0**
Mixed	0.74	0.58–0.91	**0.01**
Other Asian	0.82	0.66–1.00	0.06
Other	0.94	0.74–1.17	0.59
Pakistani	0.93	0.76–1.12	0.44
Gender			
Men	1		
Women	0.97	0.93–1.01	0.1
Age			
<45	1		
45–59	1.56	1.44–1.68	**<0.001**
60–74	2.26	2.11–2.43	**<0.001**
75+	5.65	5.31–6.03	**<0.001**
Multiple QOF conditions			
1 Condition	1		
2+ Conditions	4.69	4.48–4.91	**<0.001**
Area-level deprivation			
Least deprived Quintile 1	1		
Quintile 2	1.07	1.00–1.14	0.06
Quintile 3	1.13	1.06–1.21	**<0.001**
Quintile 4	1.11	1.04–1.19	**0**
Quintile 5	1.25	1.17–1.34	**<0.001**

Observations in all models = 250 461.

The values in bold are *p* values that are less than 0.05.

**Table 5:** Multiple logistic regression models showing the association between having a PCA record for ‘patient unsuitable’ and demographic characteristics

	Model 1			Model 2			Model 3		
	ORs	95% CI	*P*	ORs	95% CI	*P*	ORs	95% CI	*P*
Ethnicity									
White	1			1			1		
Bangladeshi	1.32	0.95–1.77	0.08	1.21	0.88–1.63	0.23	1.11	0.80–1.49	0.52
Black African	1.1	0.90–1.33	0.32	1.16	0.95–1.40	0.14	1.06	0.87–1.29	0.55
Black Caribbean	1.18	1.00–1.37	**0.05**	1.1	0.93–1.29	0.26	1.01	0.85–1.18	0.93
Black Other	1.43	1.02–1.93	**0.03**	1.41	1.01–1.91	**0.03**	1.32	0.94–1.79	0.09
Chinese	0.62	0.36–1.00	0.07	0.69	0.39–1.12	0.16	0.68	0.39–1.11	0.15
Indian	0.87	0.74–1.02	0.09	0.81	0.69–0.95	**0.01**	0.8	0.67–0.94	**0.01**
Mixed	1.09	0.86–1.36	0.45	1.1	0.87–1.36	0.43	1.05	0.83–1.31	0.68
Other Asian	1.05	0.85–1.28	0.66	1.04	0.83–1.27	0.74	1.02	0.82–1.25	0.88
Other	1.2	0.94–1.51	0.12	1.27	1.00–1.60	**0.05**	1.23	0.97–1.55	0.08
Pakistani	1.24	1.02–1.50	**0.03**	1.14	0.93–1.37	0.2	1.06	0.87–1.28	0.57
Gender									
Men	1			1			1		
Women	0.93	0.89–0.97	**<0.001**	0.94	0.90–0.98	**0.01**	0.94	0.90–0.98	**0.01**
Age									
<45	1			1			1		
45–59	1.56	1.45–1.68	**<0.001**	1.22	1.13–1.32	**<0.001**	1.23	1.14–1.33	**<0.001**
60–74	2.28	2.13–2.44	**<0.001**	1.44	1.34–1.55	**<0.001**	1.47	1.37–1.59	**<0.001**
75+	5.72	5.37–6.11	**<0.001**	3.1	2.89–3.32	**<0.001**	3.2	2.99–3.43	**<0.001**
Multiple QOF conditions									
1 Condition				1			1		
2+ Conditions				3.47	3.30–3.64	**<0.001**	3.42	3.25–3.59	**<0.001**
Area-level deprivation									
Least deprived Quintile 1							1		
Quintile 2							1.08	1.01–1.16	**0.03**
Quintile 3							1.16	1.09–1.25	**<0.001**
Quintile 4							1.2	1.12–1.29	**<0.001**
Quintile 5							1.37	1.28–1.47	**<0.001**
									

Observations in all models = 250 461. Model 1 adjusted for age and gender. Model 2 adjusted for age, gender and multiple QOF conditions. Model 3 adjusted for age, gender, multiple QOF conditions and area-level deprivation.

The values in bold are *p* values that are less than 0.05.

Black Caribbean, Black Other, Pakistani and other ethnic group patients had higher odds than white patients in the models adjusted for age, gender and multiple QOF conditions (Table 5, Models 1 and 2). These inequities were no longer evident in the final model where we adjusted for age, gender, multiple QOF conditions and area-level deprivation (Table 5, Model 3). Relative to white people, Indian people had lower odds of receiving a PCA record for ‘patient unsuitable’ in the unadjusted model (OR:0.77, 95% CI: 0.65–0.90) (Table 4) and in the fully adjusted model (OR: 0.80, 95% CI: 0.67–0.94) (Table 5, Model 3). Ethnic differences in having a PCA record for ‘patient unsuitable’ were similar for men and women (Supplementary Tables 6 and 7).

## Discussion

### Main findings of this study

In this study, we assessed patterns of PCA reporting for ‘informed dissent’ and ‘patient unsuitable’, how they varied across ethnic groups and whether they could be explained by age, gender, multiple QOF conditions and area-level deprivation. The associations between ethnicity and the two PCA reasons were in opposite directions. Most minoritized ethnic group people were less likely to have a PCA record for ‘informed dissent’. This association was significant for people of Bangladeshi, Black African, Black Caribbean, Indian, mixed, Other Asian and other ethnicity after accounting for age, gender, multiple QOF conditions and area-level deprivation. The observed ethnic inequities in PCA reporting for ‘patient unsuitable’ among people of Black Caribbean, Black Other, Pakistani and other ethnicity were explained by multiple QOF conditions and/or area-level deprivation. Only people of Indian ethnicity were significantly less likely than white people to have a PCA record for ‘patient unsuitable’.

### What is already known on this topic

The few studies that have explored ethnicity and PCA record have focused on exclusion from quality indicators for a particular condition or aggregated minoritized ethnic groups.^1^^,^^4^^,^^7^^,^^20^ Our study aligns with a previous ecological analysis showing lower levels of ‘informed dissent’ exceptions among people from minoritized ethnic groups^7^ and builds on their evidence by disaggregating ethnicity looking at PCA reasons separately. Our study also aligns with previous work showing higher rates of PCA for ‘informed dissent’ than for ‘patient unsuitable’ and higher likelihood of having a PCA record for these two reasons with increasing age, area-level deprivation and multimorbidity.^6^ In their study, women had higher odds of having a PCA record for being unsuitable, a finding which was contrary to our observations. Their conditions of interest included epilepsy, stroke, learning disability and hypothyroidism, which we did not include because these conditions did not have PCA codes for both ‘informed dissent’ and ‘patient unsuitable’.

### What this study adds

Our finding that some minoritized ethnic groups are less likely to have a PCA record for ‘informed dissent’ when compared to the white majority ethnic group raises a number of questions regarding the possible underlying mechanisms. Patients who do not agree for treatment or medical investigation can be considered to be ‘non-compliant’ or ‘non-adherent’.^21^ Racialized explanations, such as cultural and attitudinal differences, have been provided to describe why some people from minoritized ethnic groups refuse health services (e.g. clinical examinations, organ transplantation, blood transfusion, antenatal screening and immunizations), resulting in differential health outcomes and health care delivery.^22^ Such explanations ignore wider societal and structural processes (including institutional racism that has fostered mistrust of institutions^23^), which can influence how patients from minoritized ethnic groups make decisions regarding their health. Our findings suggest that they broadly accept and are compliant with the incentivized treatment.^7^ Viewed from this perspective, this finding counters dominant narratives that people from minoritized ethnic groups often refuse treatment or do not follow health recommendations due to attitudinal differences, cultural and religious beliefs.^22^

However, provision of ‘informed dissent’ allows GPs to respect patient choice,^12^ which is seen as part of a general shift toward empowerment^24^ and is increasingly recognized as crucial for preventing illness, maintaining health and improving health care provision and patient experience.^25^ This perspective represents a shift from a top-down approach to health care provision, where patients follow practitioners’ direction without question, toward an approach that is patient-centered where health care providers and patients build a sustainable partnership that can lead to mutual agreement about treatment.^21^^,^^25^ The lower likelihood of having a PCA record for ‘informed dissent’ for some minoritized ethnic group people could indicate higher levels of disempowerment.

The extant literature suggests that people from minoritized ethnic groups are more likely to experience a subjective sense of disempowerment due to cultural insensitivity and discriminatory practices within and beyond the health care setting.^26^ Lawrence and colleagues explored ethnic differences in the long-term experiences of living with psychosis and navigating mental health services.^27^ They highlight how negative expectations and experiences of these services are compounded over time, ‘creating a vicious cycle of disempowerment and mistrust that manifests for many in resistance to—or at the best passive acceptance of—intervention by mental health services’(page 5).^27^ These findings illuminate the complex relationship between (dis)empowerment and patient dissent and/or assent for people from minoritized ethnic groups. Future studies that consider the doctor–patient relationship are required to unpack this finding further.

We found that people of Black African, Black Other, Pakistani and other ethnicity were more likely to have a PCA record for ‘patient unsuitable’ compared to white people. These inequities were explained by multiple QOF conditions and area-level deprivation; people from minoritized ethnic groups are deemed more likely than their white counterparts to be unsuitable for treatment by virtue of the complex intersection of MLTCs with deprivation. Area-level deprivation and multimorbidity are inextricably linked, and many, but not all, minoritized ethnic group people have poorer health outcomes that stem from socioeconomic inequities driven by structural, institutional and interpersonal racism and discrimination.^28–30^ Recent studies also suggest that some minoritized ethnic groups have a higher prevalence of complex multimorbidity,^31^ which increases the likelihood of polypharmacy. This, in turn, increases susceptibility to inappropriate use of medications and adverse drug reactions.^32^ The link between MLTCs and patient unsuitability has also been articulated by Simpson and colleagues who suggested that patients with co-morbidities are at an increased risk of being excluded from the achievement of clinical targets because they are more likely to be intolerant to certain therapies or multiple treatments, which can result in adverse events.^33^

### Limitations of this study

The large sample size made available via CPRD Aurum allowed for the disaggregation of the minoritized ethnic group population. However, we were unable to disaggregate the white ethnic group and acknowledge that this is also a diverse population with groups, such as the Gypsy, Roma and Irish travelers, who have poor health outcomes resulting from discrimination and multiple disadvantages.^28^^,^^34^

In this study, we focused on patient-level factors and their impact on PCA patterns. However, practice-level factors, such as number of registered patients, number of GPs, practice deprivation, previous QOF performance or personal medical services contract, also impact on the rates of PCA recorded.^6^^,^^7^ Practices located in more deprived areas have a higher tendency to exclude patients for all reasons and for ‘informed dissent’.^7^ Follow-up studies are required to assess the association between ethnicity and PCAs and the extent to which key practice-level factors impact on the magnitude and direction of associations observed in this study.

We counted QOF conditions at baseline and did not consider the onset or remission of conditions or changes to QOF rules during follow-up. The conditions we included are long-term conditions, and changes would have applied across all ethnic groups. Therefore, it is unclear if this would have introduced bias, but we did not test this directly.

## Conclusions

Some view PCAs as a marker of quality because GPs who practice patient-centered, evidence-based care will inevitably have higher rates of PCAs.^35^ However, others are concerned from a public health perspective that applying PCAs can lead to a focus on patients more likely to meet targets and a corresponding reduction in the care quality given to exempted patients, thereby, leading to an increase in health inequities.^36–38^ Further, exclusions from pay-for-performance schemes means that we are less likely to have high-quality intelligence to guide improved health care. Our finding concerning the lower levels of PCA reporting for ‘informed dissent’ among 7 of the 10 minoritized ethnic groups have not only countered the prevailing narratives that suggest that people from minoritized ethnic groups refuse medical treatment, but it also illuminates the complex relationship between (dis)empowerment and ‘informed dissent’ which requires further exploration. Qualitative studies involving, for example, focus groups and in-depth interviews could illuminate the lived experience of minoritized ethnic group people and their interactions with health care professionals. Additionally, given that PCA recording is closely monitored to reduce misuse and ensure equitable care, it is important to consider that reducing rates of PCA recording for ‘informed dissent’ might come at a cost of disempowerment.

We observed inequities in PCA reporting for ‘patient unsuitability’ among people of Black African, Black Other, Pakistani and other ethnic groups, which were attenuated when we accounted for multiple QOF conditions and area-level deprivation. These groups may have unmet needs, and our analysis can inform strategies to ensure all who are eligible receive recommended standard of care. Given that exempting patients from performance targets is associated with poor disease management,^5^ and poor survival,^6^ this finding provides an insight into the mechanisms driving ethnic inequities in care, which should be addressed in the interest of preventing poor health outcomes.

## Funding

This work was supported by The Health Foundation (grant number AIMS 1874695). The study design, methods and findings were shared with members of The Health Foundation prior to publication.

## Conflict of interest

MS and JH are employed by The Health Foundation. The authors have no competing interest to declare.

## Authors’ contributions

MS, LB and BH conceptualized the study, devised the primary research questions and analysis plan. JH curated the data and provided expertise in flagging patients with relevant codes. BH conducted a review of the literature, formal statistical analysis and led the preparation of the manuscript. Output from all analyses was shared with all authors. MS, LB and MA critically reviewed, commented and edited the initial and subsequent manuscripts, providing methodological and intellectual feedback. BH revised subsequent drafts and submitted the manuscript for publication. All authors have read and agreed to the final version of the submitted manuscript. BH is the guarantor and attests that all listed authors meet authorship criteria and that no others meeting the criteria have been omitted.

## Ethical approval

The study was reviewed for ethical and methods content and approved by the CPRD team (eRAP protocol number 21_000333).

## Data availability

This study uses routinely collected individual patient data that can be obtained from CPRD subject to protocol approval via CPRD’s Research Data Governance Process. Although these data are anonymized, they are considered to be sensitive data in the UK by the Data Protection Act and, therefore, cannot be shared publicly. Information about applying to use CPRD data can be found at https://www.cprd.com/data-access. The CPRD AURUM MEDCODE IDs used to identify relevant PCA codes can be found at https://www.phpc.cam.ac.uk/pcu/research/research-groups/crmh/cprd_cam/codelists/v10-qofaurum/.

## Supplementary Material

Supplementary_Tables_revised_5_4_23_fdad104

File_of_supplementary_materials_fdad104

## Notes

**Brenda Hayanga,** Research Fellow

**Mai Stafford,** Senior Analytical Manager

**Mark Ashworth,** Professor of Primary Care

**Jay Hughes,** Data Engineer

**Laia Bécares,** Professor of Social Science and Health
