# Supplementary material for: Ethnic inequities in the patterns of personalized care adjustments for ‘informed dissent’ and ‘patient unsuitable’: a retrospective study using Clinical Practice Research Datalink
Source: J Public Health (Oxf). 2023 Jul 11;45(4):e692–701. doi: 10.1093/pubmed/fdad104 (PMC10687864; doi:10.1093/pubmed/fdad104)
Supplement: Supplementary_Tables_revised_5_4_23_fdad104 [file supplementary_tables_revised_5_4_23_fdad104.docx]

**Supplementary Table 1. Number of patients with each QOF condition**

| QOF condition | n |
| --- | --- |
| Asthma | 81028 |
| Atrial Fibrillation | 11859 |
| Cancer | 21719 |
| Coronary Heart Disease | 21075 |
| Chronic Obstructive Pulmonary Disease | 11425 |
| Dementia | 4887 |
| Depression | 90618 |
| Diabetes | 30603 |
| Heart Failure | 4530 |
| Hypertension | 91403 |
| Rheumatoid Arthritis | 3870 |
| Severe Mental Illness | 6409 |
| At least one QOF condition | 250461 |

**Supplementary Table 2. Missing ethnicity data**

|  |  | **Complete** | **Incomplete** |
| --- | --- | --- | --- |
|  | n | 250461 | 13461 |
| **Gender** | %Female | 54.3 % | 39.2 % |
|  |  |  |  |
| **Age categories** | <45 | 31.3 % | 47.1 % |
|  | 45-59 | 24.4 % | 27 % |
|  | 60-74 | 24.7 % | 19.4 % |
|  | 75+ | 19.5 % | 6.4 % |
|  |  |  |  |
| **LTCS** | 1LTCS | 64.4 % | 86.3 % |
|  | 2+LTCS | 35.6 % | 13.7 % |
|  |  |  |  |
| **Area-level**  **Deprivation** | IMD_1 | 20.9 % | 25.6 % |
|  | IMD_2 | 20.6 % | 22.6 % |
|  | IMD_3 | 19.6 % | 19.4 % |
|  | IMD_4 | 19.7 % | 18.4 % |
|  | IMD_5 | 19.2 % | 13.9 % |
| **PCA reasons** | |  |  |
|  | Informed Dissent | 7.8 % | 6.4 % |
|  | Patient Unsuitable | 3.7 % | 1.5 % |
|  |  |  |  |

**Supplementary Table 3. Demographic characteristics of sample**

|  |  | **Ethnicity** | | | | | | | | | |  | **Total** |
| --- | --- | --- | --- | --- | --- | --- | --- | --- | --- | --- | --- | --- | --- |
|  |  | **Bangladeshi** | **Black African** | **Black Caribbean** | **Black Other** | **Chinese** | **Indian** | **Mixed** | **Other Asian** | **Other** | **Pakistani** | **White** |  |
| **Gender** | Men | 629  (50.2 %) | 1705  (46.7 %) | 1626  (43.5 %) | 534  (46 %) | 323  (43.6 %) | 2608 (49.1 %) | 1266 (43.7 %) | 1487  (49 %) | 1082 (50.2 %) | 1615 (50.1 %) | 101482 (45.4 %) | **114357 (45.7 %)** |
|  |  |  |  |  |  |  |  |  |  |  |  |  |  |
|  | Women | 624  (49.8 %) | 1947  (53.3 %) | 2111  (56.5 %) | 628  (54 %) | 418  (56.4 %) | 2701 (50.9 %) | 1634 (56.3 %) | 1548 (51 %) | 1072 (49.8 %) | 1606 (49.9 %) | 121815 (54.6 %) | **136104** (**54.3 %**) |
|  |  |  |  |  |  |  |  |  |  |  |  |  |  |
| **Age categories** | <45 | 638  (50.9 %) | 1360 (37.2 %) | 969  (25.9 %) | 576  (49.6 %) | 265  (35.8 %) | 1673 (31.5 %) | 1594 (55 %) | 1129 (37.2 %) | 884 (41 %) | 1405 (43.6 %) | 67974 (30.4 %) | **78467**  (**31.3 %**) |
|  |  |  |  |  |  |  |  |  |  |  |  |  |  |
|  | 45-59 | 338  (27 %) | 1457  (39.9 %) | 1196  (32 %) | 392  (33.7 %) | 179  (24.2 %) | 1452  (27.3 %) | 732  (25.2 %) | 911  (30 %) | 642  (29.8 %) | 907  (28.2 %) | 52915  (23.7 %) | **61121**  (**24.4 %**) |
|  |  |  |  |  |  |  |  |  |  |  |  |  |  |
|  | 60-74 | 182  (14.5 %) | 610  (16.7 %) | 749  (20 %) | 123  (10.6 %) | 204  (27.5 %) | 1478  (27.8 %) | 372  (12.8 %) | 730  (24.1 %) | 412  (19.1 %) | 644  (20 %) | 56446  (25.3 %) | **61950**  (**24.7 %**) |
|  |  |  |  |  |  |  |  |  |  |  |  |  |  |
|  | 75+ | 95  (7.6 %) | 225  (6.2 %) | 823  (22 %) | 71  (6.1 %) | 93  (12.6 %) | 706  (13.3 %) | 202  (7 %) | 265  (8.7 %) | 216  (10 %) | 265  (8.2 %) | 45962  (20.6 %) | **48923**  (**19.5 %**) |
|  |  |  |  |  |  |  |  |  |  |  |  |  |  |
| **Area-level Deprivation** | Quintile 1 | 65  (5.2 %) | 124  (3.4 %) | 142  (3.8 %) | 63  (5.4 %) | 156  (21.1 %) | 863  (16.3 %) | 336  (11.6 %) | 420  (13.8 %) | 287  (13.3 %) | 227  (7 %) | 49639  (22.2 %) | **52322**  (**20.9 %**) |
|  |  |  |  |  |  |  |  |  |  |  |  |  |  |
|  | Quintile 2 | 81  (6.5 %) | 239  (6.5 %) | 244  (6.5 %) | 125  (10.8 %) | 143  (19.3 %) | 871  (16.4 %) | 378  (13 %) | 526  (17.3 %) | 315  (14.6 %) | 301  (9.3 %) | 48366  (21.7 %) | **51589**  (**20.6 %**) |
|  |  |  |  |  |  |  |  |  |  |  |  |  |  |
|  | Quintile 3 | 127  (10.1 %) | 484  (13.3 %) | 587  (15.7 %) | 156  (13.4 %) | 147  (19.8 %) | 1198  (22.6 %) | 526  (18.1 %) | 663  (21.8 %) | 403  (18.7 %) | 500  (15.5 %) | 44359  (19.9 %) | **49150**  (**19.6 %**) |
|  |  |  |  |  |  |  |  |  |  |  |  |  |  |
|  | Quintile 4 | 349  (27.9 %) | 1131  (31 %) | 1168  (31.3 %) | 336  (28.9 %) | 173  (23.3 %) | 1356  (25.5 %) | 769  (26.5 %) | 856  (28.2 %) | 578  (26.8 %) | 877  (27.2 %) | 41781  (18.7 %) | **49374**  (**19.7 %**) |
|  |  |  |  |  |  |  |  |  |  |  |  |  |  |
|  | Quintile 5 | 631  (50.4 %) | 1674  (45.8 %) | 1596  (42.7 %) | 482  (41.5 %) | 122  (16.5 %) | 1021  (19.2 %) | 891  (30.7 %) | 570  (18.8 %) | 571  (26.5 %) | 1316  (40.9 %) | 39152  (17.5 %) | **48026**  (**19.2 %**) |
|  |  |  |  |  |  |  |  |  |  |  |  |  |  |
| **Number of QOF conditions** | 1 QOF condition | 846  (67.5 %) | 2702  (74 %) | 2207  (59.1 %) | 859  (73.9 %) | 562  (75.8 %) | 3283  (61.8 %) | 2175  (75 %) | 2088  (68.8 %) | 1612  (74.8 %) | 2102  (65.3 %) | 142985  (64 %) | **161421**  (**64.4 %**) |
|  |  |  |  |  |  |  |  |  |  |  |  |  |  |
|  | 2+ QOF conditions | 407  (32.5 %) | 950  (26 %) | 1530  (40.9 %) | 303  (26.1 %) | 179  (24.2 %) | 2026  (38.2 %) | 725  (25 %) | 947  (31.2 %) | 542  (25.2 %) | 1119  (34.7 %) | 80312  (36 %) | **89040**  (**35.6 %**) |
|  |  |  |  |  |  |  |  |  |  |  |  |  |  |
| **PCA record for 'Informed Dissent'** | Present | 1172  (93.5 %) | 3425  (93.8 %) | 3508  (93.9 %) | 1077  (92.7 %) | 693  (93.5 %) | 4965  (93.5 %) | 2696  (93 %) | 2844  (93.7 %) | 2037  (94.6 %) | 2933  (91.1 %) | 205458  (92 %) | **230808**  (**92.2 %**) |
|  |  |  |  |  |  |  |  |  |  |  |  |  |  |
|  | Absent | 81  (6.5 %) | 227  (6.2 %) | 229  (6.1 %) | 85  (7.3 %) | 48  (6.5 %) | 344  (6.5 %) | 204  (7 %) | 191  (6.3 %) | 117  (5.4 %) | 288  (8.9 %) | 17839  (8 %) | **19653**  (**7.8 %**) |
|  |  |  |  |  |  |  |  |  |  |  |  |  |  |
| **PCA record for 'Patient Unsuitable'** | Present | 1210  (96.6 %) | 3544  (97 %) | 3573  (95.6 %) | 1121  (96.5 %) | 726  (98 %) | 5156  (97.1 %) | 2820  (97.2 %) | 2942  (96.9 %) | 2079  (96.5 %) | 3110  (96.6 %) | 215023  (96.3 %) | **241304**  (**96.3 %**) |
|  |  |  |  |  |  |  |  |  |  |  |  |  |  |
|  | Absent | 43  (3.4 %) | 108  (3 %) | 164  (4.4 %) | 41  (3.5 %) | 15  (2 %) | 153  (2.9 %) | 80  (2.8 %) | 93  (3.1 %) | 75  (3.5 %) | 111  (3.4 %) | 8274  (3.7 %) | **9157**  (**3.7 %**) |
|  |  |  |  |  |  |  |  |  |  |  |  |  |  |
|  | **Total** | **1253**  (**100 %**) | **3652**  (**100 %**) | **3737**  (**100 %**) | **1162**  (**100 %**) | **741**  (**100 %**) | **5309**  (**100 %**) | **2900**  (**100 %**) | **3035**  (**100 %**) | **2154**  (**100 %**) | **3221**  (**100 %**) | **223297**  (**100 %**) | **250461**  (**100 %**) |

| **Supplementary Table 4. Multiple logistic regression models showing the association between having a PCA record for ‘informed dissent’ and demographic characteristics – Men** | | | | | | | | | |
| --- | --- | --- | --- | --- | --- | --- | --- | --- | --- |
|  | **Model 1** | | | **Model 2** | | | **Model 3** | | |
|  | ORs | CI | p | ORs | CI | p | ORs | CI | p |
| ***Ethnicity*** |  |  |  |  |  |  |  |  |  |
| White | 1.00 |  |  | 1.00 |  | 1.00 |  |  |  |
| Bangladeshi | 0.70 | 0.498 – 0.943 | **0.03** | 0.65 | 0.466 – 0.884 | **0.01** | 0.62 | 0.444 – 0.844 | **0.00** |
| Black African | 0.74 | 0.614 – 0.889 | **0.00** | 0.75 | 0.622 – 0.904 | **0.00** | 0.72 | 0.591 – 0.860 | **<0.001** |
| Black Caribbean | 0.78 | 0.640 – 0.937 | **0.01** | 0.76 | 0.623 – 0.914 | **0.01** | 0.72 | 0.595 – 0.873 | **0.00** |
| Black Other | 0.94 | 0.682 – 1.253 | 0.67 | 0.96 | 0.694 – 1.281 | 0.77 | 0.91 | 0.665 – 1.227 | 0.57 |
| Chinese | 0.98 | 0.652 – 1.422 | 0.93 | 1.08 | 0.717 – 1.570 | 0.69 | 1.08 | 0.717 – 1.570 | 0.69 |
| Indian | 0.85 | 0.732 – 0.979 | **0.03** | 0.81 | 0.699 – 0.936 | **0.01** | 0.81 | 0.695 – 0.931 | **0.00** |
| Mixed | 1.01 | 0.826 – 1.218 | 0.94 | 1.01 | 0.829 – 1.226 | 0.90 | 0.99 | 0.810 – 1.198 | 0.92 |
| Other Asian | 0.84 | 0.688 – 1.009 | 0.07 | 0.83 | 0.683 – 1.005 | 0.06 | 0.83 | 0.677 – 0.996 | 0.05 |
| Other | 0.60 | 0.456 – 0.768 | **<0.001** | 0.62 | 0.474 – 0.798 | **<0.001** | 0.61 | 0.463 – 0.781 | **<0.001** |
| Pakistani | 1.04 | 0.872 – 1.224 | 0.68 | 0.99 | 0.833 – 1.173 | 0.93 | 0.95 | 0.801 – 1.129 | 0.59 |
| ***Age*** |  |  |  |  |  |  |  |  |  |
| <45 | 1.00 |  |  | 1.00 |  |  | 1.00 |  |  |
| 45-59 | 1.41 | 1.334 – 1.485 | **<0.001** | 1.18 | 1.120 – 1.251 | **<0.001** | 1.19 | 1.126 – 1.257 | **<0.001** |
| 60-74 | 1.05 | 0.991 – 1.108 | 0.10 | 0.75 | 0.705 – 0.794 | **<0.001** | 0.76 | 0.715 – 0.806 | **<0.001** |
| 75+ | 0.86 | 0.807 – 0.920 | **<0.001** | 0.54 | 0.506 – 0.584 | **<0.001** | 0.56 | 0.517 – 0.597 | **<0.001** |
| ***MLTCS*** |  |  |  |  |  |  |  |  |  |
| 1 LTC |  |  |  | 1.00 |  |  | 1.00 |  |  |
| 2+ LTCS |  | | | 2.30 | 2.200 – 2.410 | **<0.001** | 2.28 | 2.181 – 2.390 | **<0.001** |
| ***Deprivation: IMD*** |  |  |  |  |  |  |  |  |  |
| Quintile 1 |  |  |  |  |  |  | 1.00 |  |  |
| Quintile 2 |  | | |  | | | 1.01 | 0.945 – 1.079 | 0.78 |
| Quintile 3 |  |  |  |  | | | 1.04 | 0.974 – 1.113 | **0.241** |
| Quintile 4 |  |  |  |  |  |  | 1.09 | 1.021 – 1.166 | **0.01** |
| Quintile 5 |  |  |  |  |  |  | 1.18 | 1.100 – 1.255 | **<0.001** |
| Observations : 114357  Model 1 Adjusted for age  Model 2 Adjusted for age and multiple QOF conditions Model 3 Adjusted for age, multiple QOF conditions, and area level deprivation | | | | | | | | | |

| **Supplementary Table 5. Multiple logistic regression models showing the association between having a PCA record for ‘informed dissent’ and demographic characteristics – Women** | | | | | | | | | |
| --- | --- | --- | --- | --- | --- | --- | --- | --- | --- |
|  | **Model 1** | | | **Model 2** | | | **Model 3** | | |
|  | ORs | CI | p | ORs | CI | p | ORs | CI | p |
| ***Ethnicity*** |  |  |  |  |  |  |  |  |  |
| White | 1.00 |  |  | 1.00 |  | 1.00 |  |  |  |
| Bangladeshi | 0.89 | 0.635 – 1.210 | 0.48 | 0.84 | 0.597 – 1.143 | 0.29 | 0.78 | 0.557 – 1.067 | 0.14 |
| Black African | 0.71 | 0.577 – 0.860 | **0.00** | 0.74 | 0.599 – 0.895 | **0.00** | 0.69 | 0.562 – 0.841 | **<0.001** |
| Black Caribbean | 0.71 | 0.579 – 0.849 | **<0.001** | 0.66 | 0.542 – 0.797 | **<0.001** | 0.62 | 0.509 – 0.748 | **<0.001** |
| Black Other | 0.84 | 0.600 – 1.151 | 0.31 | 0.82 | 0.582 – 1.123 | 0.24 | 0.78 | 0.555 – 1.071 | 0.14 |
| Chinese | 0.64 | 0.393 – 0.972 | **0.05** | 0.70 | 0.432 – 1.074 | 0.13 | 0.70 | 0.430 – 1.068 | 0.12 |
| Indian | 0.70 | 0.588 – 0.828 | **<0.001** | 0.68 | 0.567 – 0.800 | **<0.001** | 0.67 | 0.560 – 0.790 | **<0.001** |
| Mixed | 0.76 | 0.607 – 0.932 | **0.01** | 0.76 | 0.610 – 0.940 | **0.01** | 0.74 | 0.591 – 0.911 | **0.01** |
| Other Asian | 0.65 | 0.513 – 0.816 | **<0.001** | 0.65 | 0.511 – 0.816 | **<0.001** | 0.64 | 0.506 – 0.808 | **<0.001** |
| Other | 0.70 | 0.528 – 0.908 | **0.01** | 0.74 | 0.560 – 0.966 | **0.03** | 0.73 | 0.550 – 0.949 | **0.02** |
| Pakistani | 1.20 | 1.001 – 1.424 | **0.04** | 1.13 | 0.940 – 1.342 | 0.19 | 1.07 | 0.892 – 1.275 | 0.46 |
| ***Age*** |  |  |  |  |  |  |  |  |  |
| <45 | 1.00 |  |  | 1.00 |  |  | 1.00 |  |  |
| 45-59 | 1.26 | 1.195 – 1.335 | **<0.001** | 1.07 | 1.014 – 1.135 | **0.02** | 1.08 | 1.021 – 1.144 | **0.01** |
| 60-74 | 1.12 | 1.055 – 1.184 | **<0.001** | 0.80 | 0.749 – 0.845 | **<0.001** | 0.81 | 0.762 – 0.860 | **<0.001** |
| 75+ | 1.11 | 1.044 – 1.176 | **0.001** | 0.68 | 0.637 – 0.723 | **<0.001** | 0.69 | 0.651 – 0.740 | **<0.001** |
| ***MLTCS*** |  |  |  |  |  |  |  |  |  |
| 1 LTC |  |  |  | 1.00 |  |  | 1.00 |  |  |
| 2+ LTCS |  | | | 2.83 | 2.701 – 2.955 | **<0.001** | 2.79 | 2.670 – 2.921 | **<0.001** |
| ***Deprivation: IMD*** |  |  |  |  |  |  |  |  |  |
| Quintile 1 |  |  |  |  |  |  | 1.00 |  |  |
| Quintile 2 |  | | |  | | | 1.00 | 0.932 – 1.066 | 0.93 |
| Quintile 3 |  |  |  |  | | | 1.05 | 0.984 – 1.125 | 0.14 |
| Quintile 4 |  |  |  |  |  |  | 1.11 | 1.034 – 1.182 | **0.00** |
| Quintile 5 |  |  |  |  |  |  | 1.23 | 1.151 – 1.314 | **<0.001** |
| Observations : 136104 Model 1 Adjusted for age  Model 2 Adjusted for age and multiple QOF conditions Model 3 Adjusted for age, multiple QOF conditions, and area level deprivation | | | | | | | | | |

| **Supplementary Table 6. Multiple logistic regression models showing the association between having a PCA record for ‘patient unsuitable’ and demographic characteristics – Men** | | | | | | | | | |
| --- | --- | --- | --- | --- | --- | --- | --- | --- | --- |
|  | **Model 1** | | | **Model 2** | | | **Model 3** | | |
|  | ORs | CI | p | ORs | CI | p | ORs | CI | p |
| ***Ethnicity*** |  |  |  |  |  |  |  |  |  |
| White | 1.00 |  |  | 1.00 |  | 1.00 |  |  |  |
| Bangladeshi | 1.55 | 1.024 – 2.241 | **0.03** | 1.41 | 0.928 – 2.042 | 0.09 | 1.28 | 0.842 – 1.857 | 0.22 |
| Black African | 1.03 | 0.772 – 1.352 | 0.82 | 1.07 | 0.797 – 1.401 | 0.64 | 0.96 | 0.718 – 1.264 | 0.80 |
| Black Caribbean | 1.34 | 1.063 – 1.668 | **0.01** | 1.28 | 1.011 – 1.593 | **0.03** | 1.16 | 0.912 – 1.442 | 0.22 |
| Black Other | 1.51 | 0.954 – 2.270 | 0.06 | 1.56 | 0.983 – 2.354 | **0.04** | 1.43 | 0.896 – 2.150 | 0.11 |
| Chinese | 0.53 | 0.210 – 1.094 | 0.13 | 0.60 | 0.234 – 1.231 | 0.21 | 0.60 | 0.234 – 1.228 | 0.21 |
| Indian | 0.95 | 0.758 – 1.177 | 0.66 | 0.88 | 0.702 – 1.093 | 0.27 | 0.87 | 0.691 – 1.077 | 0.21 |
| Mixed | 1.06 | 0.749 – 1.454 | 0.73 | 1.06 | 0.749 – 1.460 | 0.72 | 1.02 | 0.715 – 1.395 | 0.93 |
| Other Asian | 0.85 | 0.605 – 1.152 | 0.32 | 0.83 | 0.592 – 1.131 | 0.26 | 0.82 | 0.580 – 1.109 | 0.21 |
| Other | 1.26 | 0.903 – 1.702 | 0.16 | 1.35 | 0.964 – 1.826 | 0.07 | 1.29 | 0.921 – 1.745 | 0.12 |
| Pakistani | 1.32 | 1.012 – 1.684 | **0.03** | 1.21 | 0.929 – 1.551 | 0.14 | 1.12 | 0.856 – 1.432 | 0.40 |
| ***Age*** |  |  |  |  |  |  |  |  |  |
| <45 | 1.00 |  |  | 1.00 |  |  | 1.00 |  |  |
| 45-59 | 1.64 | 1.476 – 1.817 | **<0.001** | 1.24 | 1.113 – 1.377 | **<0.001** | 1.25 | 1.126 – 1.392 | **<0.001** |
| 60-74 | 2.12 | 1.920 – 2.337 | **<0.001** | 1.28 | 1.152 – 1.416 | **<0.001** | 1.32 | 1.186 – 1.460 | **<0.001** |
| 75+ | 4.67 | 4.259 – 5.133 | **<0.001** | 2.41 | 2.181 – 2.672 | **<0.001** | 2.52 | 2.279 – 2.795 | **<0.001** |
| ***MLTCS*** |  |  |  |  |  |  |  |  |  |
| 1 LTC |  |  |  | 1.00 |  |  | 1.00 |  |  |
| 2+ LTCS |  | | | 3.38 | 3.144 – 3.633 | **<0.001** | 3.32 | 3.093 – 3.574 | **<0.001** |
| ***Deprivation: IMD*** |  |  |  |  |  |  |  |  |  |
| Quintile 1 |  |  |  |  |  |  | 1.00 |  |  |
| Quintile 2 |  | | |  | | | 1.12 | 1.012 – 1.237 | **0.03** |
| Quintile 3 |  |  |  |  | | | 1.20 | 1.083 – 1.324 | **<0.001** |
| Quintile 4 |  |  |  |  |  |  | 1.20 | 1.084 – 1.329 | **<0.001** |
| Quintile 5 |  |  |  |  |  |  | 1.48 | 1.339 – 1.633 | **<0.001** |
| Observations : 114357  Model 1 Adjusted for age  Model 2 Adjusted for age and multiple QOF conditions Model 3 Adjusted for age, multiple QOF conditions, and area level deprivation | | | | | | | | | |

| **Supplementary Table 7. Multiple logistic regression models showing the association between having a PCA record for ‘patient unsuitable’ and demographic characteristics – Women** | | | | | | | | | |
| --- | --- | --- | --- | --- | --- | --- | --- | --- | --- |
|  | **Model 1** | | | **Model 2** | | | **Model 3** | | |
|  | ORs | CI | p | ORs | CI | p | ORs | CI | p |
| ***Ethnicity*** |  |  |  |  |  |  |  |  |  |
| White | 1.00 |  |  | 1.00 |  | 1.00 |  |  |  |
| Bangladeshi | 1.03 | 0.600 – 1.648 | 0.90 | 0.96 | 0.557 – 1.537 | 0.88 | 0.89 | 0.515 – 1.424 | 0.65 |
| Black African | 1.17 | 0.884 – 1.520 | 0.25 | 1.25 | 0.940 – 1.621 | 0.11 | 1.16 | 0.876 – 1.514 | 0.28 |
| Black Caribbean | 1.05 | 0.830 – 1.308 | 0.68 | 0.96 | 0.762 – 1.203 | 0.76 | 0.90 | 0.709 – 1.124 | 0.36 |
| Black Other | 1.35 | 0.820 – 2.076 | 0.21 | 1.28 | 0.776 – 1.980 | 0.30 | 1.21 | 0.735 – 1.879 | 0.42 |
| Chinese | 0.71 | 0.338 – 1.298 | 0.31 | 0.79 | 0.373 – 1.446 | 0.48 | 0.78 | 0.368 – 1.428 | 0.46 |
| Indian | 0.78 | 0.607 – 0.991 | **0.05** | 0.74 | 0.571 – 0.935 | **0.02** | 0.72 | 0.559 – 0.916 | **0.01** |
| Mixed | 1.13 | 0.815 – 1.511 | 0.45 | 1.13 | 0.819 – 1.525 | 0.43 | 1.09 | 0.787 – 1.467 | 0.59 |
| Other Asian | 1.26 | 0.943 – 1.640 | 0.10 | 1.25 | 0.939 – 1.640 | 0.11 | 1.23 | 0.921 – 1.610 | 0.14 |
| Other | 1.13 | 0.784 – 1.572 | 0.49 | 1.17 | 0.808 – 1.631 | 0.38 | 1.15 | 0.793 – 1.601 | 0.44 |
| Pakistani | 1.16 | 0.849 – 1.532 | 0.34 | 1.05 | 0.773 – 1.400 | 0.73 | 0.99 | 0.729 – 1.322 | 0.97 |
| ***Age*** |  |  |  |  |  |  |  |  |  |
| <45 | 1.00 |  |  | 1.00 |  |  | 1.00 |  |  |
| 45-59 | 1.46 | 1.312 – 1.634 | **<0.001** | 1.18 | 1.057 – 1.320 | **0.003** | 1.19 | 1.066 – 1.331 | **0.002** |
| 60-74 | 2.44 | 2.211 – 2.702 | **<0.001** | 1.61 | 1.448 – 1.780 | **<0.001** | 1.63 | 1.474 – 1.813 | **<0.001** |
| 75+ | 6.75 | 6.178 – 7.396 | **<0.001** | 3.78 | 3.441 – 4.160 | **<0.001** | 3.87 | 3.525 – 4.264 | **<0.001** |
| ***MLTCS*** |  |  |  |  |  |  |  |  |  |
| 1 LTC |  |  |  | 1.00 |  |  | 1.00 |  |  |
| 2+ LTCS |  | | | 3.59 | 3.352 – 3.839 | **<0.001** | 3.54 | 3.311 – 3.793 | **<0.001** |
| ***Deprivation: IMD*** |  |  |  |  |  |  |  |  |  |
| Quintile 1 |  |  |  |  |  |  | 1.00 |  |  |
| Quintile 2 |  | | |  | | | 1.05 | 0.954 – 1.148 | **0.034** |
| Quintile 3 |  |  |  |  | | | 1.13 | 1.032 – 1.242 | **0.009** |
| Quintile 4 |  |  |  |  |  |  | 1.19 | 1.082 – 1.304 | **<0.001** |
| Quintile 5 |  |  |  |  |  |  | 1.27 | 1.157 – 1.395 | **<0.001** |
| Observations : 136104 Model 1 Adjusted for age  Model 2 Adjusted for age and multiple QOF conditions Model 3 Adjusted for age, multiple QOF conditions, and area level deprivation | | | | | | | | | |
